# Effects of adaptive immune cell therapy on the immune cell profile in patients with advanced gastric cancer

**DOI:** 10.1002/cam4.3152

**Published:** 2020-06-11

**Authors:** Miyabi Miura, Eishiro Mizukoshi, Tomomi Hashiba, Masaaki Kitahara, Tomoharu Miyashita, Takafumi Mochizuki, Shigenori Goto, Takashi Kamigaki, Rishu Takimoto, Taro Yamashita, Yoshio Sakai, Tatsuya Yamashita, Masao Honda, Shuichi Kaneko

**Affiliations:** ^1^ Department of Gastroenterology Graduate School of Medicine Kanazawa University Kanazawa City Japan; ^2^ Kanazawa Advanced Medical Center Kanazawa City Japan; ^3^ Seta Clinic Group Department of Next‐Generation Cell and Immune Therapy Juntendo University School of Medicine Tokyo Japan

**Keywords:** gastric cancer, immune cell profile, immunotherapy, PD‐1, αβT‐cell therapy

## Abstract

**Background:**

Immunotherapy for cancer patients has been the subject of attention in recent years. In this study, we investigated whether αβT‐cell therapy causes changes in the host's immune cell profile, and if so, the effect of these changes on prognosis.

**Methods:**

Peripheral blood mononuclear cells (PBMCs) from 30 gastric cancer patients who had completed one course of αβT‐cell therapy were analyzed. The peripheral blood immune cell profile was established using PBMCs by counting the frequency of CD4+ helper T cells, CD8+ killer T cells, regulatory T cells (Tregs), and myeloid‐derived suppressor cells and measuring the expression of their surface markers. The changes after treatment and their association with response to treatment were investigated.

**Results:**

Immune cell profiles changed greatly after treatment. The frequency of CD4+ helper T cells decreased, but that of CD8+ killer T cells increased. The frequency of programmed cell death 1 (PD‐1)+ effector Tregs increased significantly, but only in the non‐progressive disease (non‐PD) group, in which it was significantly higher compared with the PD group. Patients in whom the frequency of PD‐1+ effector Tregs increased had a significantly better prognosis than those in whom it decreased.

**Conclusion:**

Our results suggested that αβT‐cell therapy changes the host's immune cell profile, and an increase in PD‐1+ effector Tregs may help improve prognosis.

## Graphical abstract

Our results suggested that αβT‐cell therapy changes the host's immune cell profile. An increase in PD‐1+ effector Tregs may help improve prognosis of advanced gastric cancer.

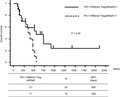

## 1 INTRODUCTION

Cancer immunotherapy is a promising fourth treatment for cancer after surgery, drug therapy, and radiotherapy. It encompasses treatments with cytokines, antibodies, immune checkpoint inhibitors, and immune cells. The successful clinical use of immune checkpoint inhibitors and T cells into which a chimeric antigen receptor (CAR) gene has been inserted (CAR‐T cells) has led to a reaffirmation of the importance of the host's immune cells, primarily T cells, in recognizing and attacking tumor cells. Unlike existing therapies such as anticancer agents and molecular targeted drugs, which target the cancer itself, these new treatment methods are revolutionary in the sense that they treat cancer by targeting the host's immune system, a paradigm shift that is leading to the formation of new cancer treatment strategies.

Gastric cancer is the fifth most common form of cancer worldwide, and the third most common cause of death.^1^ In recent years, the number of patients has been decreasing thanks to *Helicobacter pylori* eradication therapy and early discovery of cancer due to improvements in endoscopic techniques. In addition to the mainstream treatments of surgery and chemotherapy, molecular targeted drugs and immune checkpoint inhibitors have also been developed to treat gastric cancer, and these have been significantly transforming its treatment in recent years. The molecular targeted drugs used include trastuzumab and ramucirumab, and the anti‐programmed cell death 1 (PD‐1) antibody nivolumab is also used as an immune checkpoint inhibitor. Many other immune checkpoint inhibitors are currently under development, including the anti‐PD‐1 antibody pembrolizumab, the anti‐PD ligand 1 (PD‐L1) antibodies avelumab, atezolizumab, and durvalumab, and the anti‐cytotoxic T‐lymphocyte–associated protein 4 (CTLA‐4) antibody ipilimumab. In terms of the effectiveness of immune checkpoint inhibitors against advanced gastric cancer; however, only a limited number of patients respond to treatment, and as yet, the improvement in their prognosis is insufficient.

One reason for this is believed to be the mechanisms by which cancers evade the immune system. Some cancers use immunosuppressive mechanisms such as regulatory T cells (Tregs) and immune checkpoint molecules to grow, whereas in others, the cancer cells themselves decrease highly immunogenic antigens to escape from the immune system. Cancer cells form a cancer microenvironment around them that differs from normal tissues, and the release of angiogenic factors promotes angiogenesis in these areas, with killer T cells and regulatory T cells infiltrating these sites via the newly formed vessels.^2^ Among the activated Tregs that infiltrate cancer microenvironments, cells with high chemokine receptor (CCR)4 expression that intensify antitumor immunity via anti‐CCR4 antibodies have been reported in malignant melanoma,^3^ and individual cancer patients possess specific cancer microenvironments and immune cell profiles.

Immune cell profiles are thus believed to affect the prognosis of patients and their response to immunotherapy. However, this has yet to be fully investigated in patients with advanced gastric cancer.

In this study, we carried out immune cell profiling of patients with advanced gastric cancer following immune cell therapy, with the aim of identifying factors associated with their condition, prognosis, and response to immune cell therapy.

## 2 PATIENTS AND METHODS

### 2.1 Patient population

The study subjects were 30 gastric cancer patients (23 men and seven women, mean age 61.6 ± 10.0 years) who completed one course of αβT‐cell therapy at our affiliated facility between April 2010 and December 2016 (Consort diagram is shown in Figure S1). All patients were performance status^4^ 0 or 1, and stage III or IV according to the TNM classification. Of those patients who underwent response evaluation, 13 were classed as progressive disease (PD) and 10 as non‐PD, and the association between their immune cell profile and prognosis was analyzed.

This study was conducted in accordance with the Declaration of Helsinki and approved by the hospital ethics committee. Written informed consent was obtained from all patients before the start of αβT‐cell therapy.

### 2.2 Laboratory tests and imaging

Tumor marker assays for carcinoembryonic antigen (CEA), carbohydrate antigen 19‐9 (CA19‐9), and α‐fetoprotein (AFP) (FALCO) were carried out using patient serum before the start of αβT‐cell therapy and after the administration of one course. Serum CEA and serum AFP levels were measured with a chemiluminescence immunoassay, and the serum CA19‐9 level with an electrochemiluminescence immunoassay.

Imaging for response assessment was carried out with positron emission tomography computed tomography (PET‐CT) before the start of αβT‐cell therapy and after the administration of one course, with evaluation carried out using the New Response Evaluation Criteria in Solid Tumours (Revised RECIST Guideline; version 1.1). Whether or not the RECIST 1.1 standards are the most appropriate method of assessing response to immunotherapy is still a matter of debate.^5^ In light of the issue of pseudoprogression, in this study we used PET‐CT for response evaluation as the method that most accurately evaluates tumor viability.^6^ Complete response (CR) was defined as the disappearance of all target lesions for which fluorodeoxyglucose uptake had been detected on PET‐CT; partial response (PR) as a decrease of ≥30% in the sum of the diameters of the target lesions compared with the sum of their diameters at baseline; PD as an increase of ≥20% in the sum of the diameters of the target lesions compared with the smallest sum of their diameters measured during the course of treatment, as well as an absolute increase of ≥5 mm in the sum of their diameters; and stable disease (SD) as neither a decrease in size corresponding to PR nor an increase in size corresponding to PD compared with the smallest sum of their diameters measured during the course of treatment. CR, PR, and SD were defined as non‐PD, and the treated patients were divided into a PD group and a non‐PD group.

### 2.3 αβT‐cell therapy

Lymphocytes were isolated from 24 mL peripheral blood drawn from patients, and activated by culturing with anti‐CD3 monoclonal antibody (Jansen‐Kyowa). They were then cultured for 14 days with interleukin‐2 until the cell count was ≥0.3 × 10^9^, and αβT cells with a viable cell rate of ≥80%, endotoxin test results <0.25 EU/mL, and that passed sterility testing were returned to the patient's body. This was performed six times at 2‐week intervals to complete one course. The cultured lymphocytes consisted of mainly CD8+ or CD4+ T cells (αβT‐cells) with small percentages of natural killer cells and γδT cells.

### 2.4 Preparation of peripheral blood mononuclear cells

Peripheral blood mononuclear cell (PBMCs) were isolated according to a previously established method from 29 mL peripheral blood drawn from patients before the start of αβT‐cell therapy and after the administration of one course.^7^ These PBMCs were suspended in CELLBANKER 1 (ZENOAQ) containing 80% fetal calf serum and 10% dimethyl sulfoxide, and stored frozen at −150°C in a liquid nitrogen tank before analysis for this study.

### 2.5 Analysis of peripheral immune cell profiles

To determine the frequency of immune cells, multi‐color fluorescence‐activated cell sorting (FACS) analysis was performed using the following antibodies: anti‐CD3, CD4, CD8, CD14, CD15, CD25, CD80, CD45RA, HLA‐DR, FoxP3, CTLA‐4, PD‐1, PD‐L1, CCR4, CCD6, CXCR3, 4‐1BB, and OX40 (Becton Dickinson). Flow cytometry was done using the Becton Dickinson FACSAria II system.

### 2.6 Statistical analysis

Data are expressed as the mean ± standard deviation. Fisher's exact test (two‐sided *P‐*value) and the unpaired Student's t test were used to analyze the clinical factors of the patients. A *P* value of <.05 was considered to be significant, and all the tests were two‐sided.

## 3 RESULTS

### 3.1 Patient profile

Table 1 shows the clinical attributes of the gastric cancer patients analyzed in this study. The 30 patients comprised 23 men and seven women with a mean age of 61.6 ± 10 years at the start of immunotherapy, and their performance status was 0 or 1 in all cases. Following the Union International for Cancer Control TNM classification (7th edition), the tumor stage was III or IV in all cases (three stage III, 27 stage IV). In terms of the Japanese Classification of Gastric Carcinoma (14th edition),^8^ the histological classification was papillary adenocarcinoma (pap) in three cases, well‐differentiated tubular adenocarcinoma (tub1) in zero, moderately differentiated tubular adenocarcinoma (tub2) in eight, poorly differentiated adenocarcinoma (por) in 14, signet‐ring cell carcinoma (sig) plus mucinous adenocarcinoma (muc) in two, and unknown in three. Distant metastasis was present in 26 cases, and 25 patients had undergone previous treatment (surgery plus chemotherapy in 11 cases, surgery alone in four, chemotherapy alone in eight, and surgery plus chemotherapy plus radiotherapy in two), whereas 29 were undergoing combination therapy (surgery plus chemotherapy in two cases, chemotherapy in 27, and radiotherapy in zero). One patient underwent immunotherapy alone. The chemotherapy administered as pretreatment comprised Tegafur/Gimeracil/Oteracil/Potassium (S‐1) in five cases, S‐1 plus cisplatin (CDDP) in six, S‐1 plus docetaxel (DTX) in one, S‐1 plus irinotecan (CPT‐11) in one, paclitaxel (PTX) in one, S‐1 plus CDDP plus lentinan in one, CPT‐11 in one, DTX in one, DTX plus doxifluridine in one, trastuzumab (HER) plus capecitabine plus cisplatin (XP) in one, PTX plus ramucirumab in one, and docetaxel plus cisplatin plus S‐1 (DCS) in one (Table S1). The chemotherapy administered in combination with αβT‐cell therapy comprised S‐1 plus CDDP therapy in 11 cases, PTX monotherapy in four, S‐1 monotherapy in three, CPT‐11 plus CDDP therapy in two, CPT‐11 monotherapy in two, S‐1 plus oxaliplatin (SOX) in two, S‐1 plus DTX in two, HER plus Xeloda/PTX in one, HER plus DCS in one, and HER plus XP in one (Table S2). The chemotherapy administered in combination with αβT‐cell therapy was first‐line therapy in 17 cases, second‐line in six, third‐line in two, fourth‐line in one, and fifth‐line in three (Table S3).

**Table 1:** Patients’ characteristics

	All cases (n = 30)
Age (range)	61.6 (39‐78)
Sex, M/F	23/7
PS, 0/1/2‐4	19/11/0
TNM stage, I/II/III/IV	0/0/3/27
Histological differentiation, pap/tub1/tub2/por/sig + muc/unknown	3/0/8/14/2/3
Distant metastasis, Yes/No	26/4
Prior treatment, Yes/No	25/5
Surgery, Yes/No	17/13
Chemotherapy, Yes/No	21/9
Radiation therapy, Yes/No	2/28
Combined therapy, Yes/No	29/1
Surgery, Yes/No	2/28
Chemotherapy, Yes/No	29/1
Radiation therapy, Yes/No	0/30
Clinical response, CR/PR/SD/PD/NE	2/1/7/13/7

Abbreviations: CR, complete response; muc, mucinous adenocarcinoma; NE, not evaluable; pap, papillary adenocarcinoma; PD, progressive disease; por, poorly differentiated adenocarcinoma; PR, partial response; PS, Performance status; SD, stable disease; sig, signet‐ring cell carcinoma; TNM, tumor‐node‐metastasis; tub1, well differentiated tubular adenocarcinoma; tub2, moderately differentiated tubular adenocarcinoma.

### 3.2 Treatment outcomes of αβT‐cell therapy

The response to αβT‐cell therapy was CR in two cases, PR in one, SD in seven, PD in 13, and not evaluable in seven (Table 1). Overall survival (OS) was defined as the period from the date αβT‐cell therapy was started until the date of death or final confirmation of survival. Mean OS for all patients was 500.6 days, and median OS was 469 days. There were no statistically significant differences in clinical attributes between the PD group and the CR plus PR plus SD (non‐PD) group (Table 2). Mean and median OS were 338.8 and 298 days, respectively, in the PD group and 627.6 and 504 days, respectively, in the non‐PD group, with non‐PD patients tending to have a better prognosis, although this difference was not statistically significant due to the small number of cases (Figure S2). There were no severe adverse events as a result of αβT‐cell therapy. Mild adverse events were recorded in six patients (20.0%), but all of these improved spontaneously (Table S4).

**Table 2:** Patients’ characteristics in PD group and non PD group

	PD (n = 13)	Non PD (n = 10)	*P* value
Age (range)	63.2 (56‐70)	62.4 (55‐74)	NS
Sex, M/F	10/3	8/2	NS
PS, 0/1/2‐4	9/4/0	6/4/0	NS
TNM stage, I/II/III/IV	0/0/0/13	0/0/0/10	NS
Histological differentiation			
pap/tub1/tub2/por/sig + muc/unknown	3/0/3/5/0/2	0/0/2/6/1/1	NS
Distant metastasis, Yes/No	13/0	10/0	NS
Prior treatment, Yes/No	12/1	7/3	NS
Surgery, Yes/No	6/7	6/4	NS
Chemotherapy, Yes/No	11/2	6/4	NS
Radiation therapy, Yes/No	0/13	1/9	NS
Combined therapy, Yes/No	13/0	10/0	NS
Surgery, Yes/No	0/13	0/10	NS
Chemotherapy, Yes/No	13/0	10/0	NS
Radiation therapy, Yes/No	0/13	0/10	NS

Abbreviations: muc, mucinous adenocarcinoma; pap, papillary adenocarcinoma; por, poorly differentiated adenocarcinoma; PS, Performance status; sig, signet‐ring cell carcinoma; TNM, tumor‐node‐metastasis; tub1, well differentiated tubular adenocarcinoma; tub2, moderately differentiated tubular adenocarcinoma.

### 3.3 Measurement of immune cell profiles in peripheral blood

**Figure 1:**
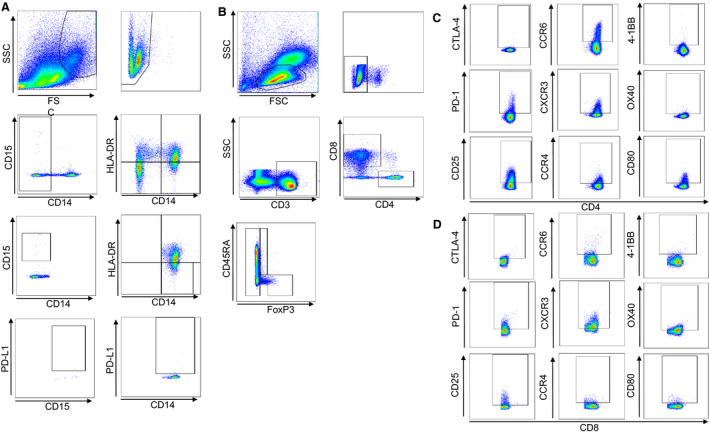
Peripheral blood immune cell profiles. A, The frequencies of MDSCs with expression of cell surface markers were measured by multi‐color FACS analysis using the following antibodies: anti‐CD14, CD15, and HLA‐DR. B, The frequencies of CD4+ T cells, CD8+ T cells, and Tregs with expression of cell surface markers were measured by multi‐color FACS analysis using the following antibodies: anti‐CD3, CD4, CD8, CD45RA, and FoxP3. Effector Tregs were defined as CD3+, CD4+, CD45RA−, and FoxP3‐high cells; naïve Tregs were defined as CD3+, CD4+, CD45RA+, and FoxP3‐low cells; and helper T cells were defined as CD3+, CD4+, CD45RA+/−, and FoxP3−/low cells. C, Expression levels of CD25, CTLA‐4, PD‐1, CCR4, CXCR3, CCR6, CD80, OX40, and 4‐1BB were measured in Tregs and helper T cells. D, Expression levels of CD25, CTLA‐4, PD‐1, CCR4, CXCR3, CCR6, CD80, OX40, and 4‐1BB were also measured in CD8+ T cells. FSC, forward scatter; SSC, side scatter

We next investigated immune cell profiles in peripheral blood. In this study, myeloid‐derived suppressor cells (MDSCs) in peripheral blood were divided into CD14 + CD15− MDSCs and CD14‐ CD15+ MDSCs according to their levels of expression of CD14, CD15, and HLA‐DR, and the frequency of each type was measured. Then the PD‐L1 expression level in each fraction was measured (Figure 1A). For T cells, CD8+ T cells and CD4+ T cells were isolated, and from the CD4+ T cells, CD45RA− effector Tregs that strongly expressed FoxP3, CD45RA+ naïve Tregs that weakly expressed FoxP3, and CD4+ cells that were negative for FoxP3 (defined as helper T cells) were further isolated for investigation (Figure 1B), as previously reported by Nishikawa et al^9^ The levels of expression of CTLA‐4, PD‐1, CD25, CCR6, CXCR3, CCR4, 4‐1BB, OX40, and CD80 in the different T‐cell fractions were also measured (Figure 1C,D).

### 3.4 Change in immune cell profile before and after treatment

**Figure 2:**
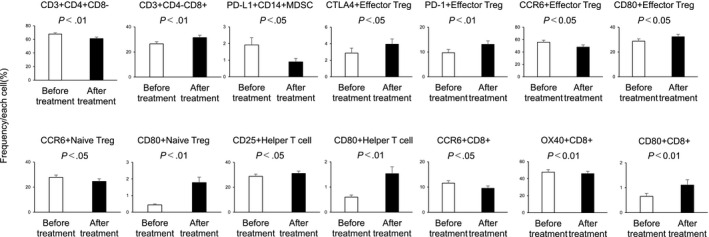
Frequencies of peripheral blood immune cells with each profile in patients before αβT‐cell therapy and after αβT‐cell therapy. Only significant results are shown. The data are the mean ± SD

We investigated whether or not the immune cell profile changed after the immune cell therapy. Figure 2 shows those immune cells of the peripheral blood immune cell profile for which statistically significant changes were evident after treatment (the other results are shown in Figure S3).

The mean frequencies of the following cells decreased significantly after treatment compared with before treatment: CD3+ CD4+CD8–(61.20% ± 2.09% vs 67.81% ± 1.88%; *P* < .01), PD‐L1 + CD14+ MDSCs (0.89% ± 0.21% vs 1.91% ± 0.44%; *P* < .05), CCR6 + effector Tregs (47.94% ± 3.63% vs 55.73% ± 3.41%; *P* < .05), CCR6+ naïve Tregs (24.50% ± 2.13% vs 27.81% ± 1.82%; *P* < .05), CCR6 + CD8 (9.49% ± 0.99% vs 11.56% ± 0.95%; *P* < .05), and OX40 + CD8+ (45.80% ± 2.82% vs 47.65% ± 2.91%; *P* < .01). Conversely, the mean frequencies of the following cells increased significantly after treatment compared with before treatment: CD3+ CD4‐CD8+ (31.48% ± 1.85% vs 26.46% ± 1.68%; *P* < .01), CTLA‐4+ effector Tregs (3.95% ± 0.64% vs 2.87% ± 0.60%; *P* < .05), PD‐1+ effector Tregs (13.06% ± 1.41% vs 9.68% ± 1.32%; *P* < .01), CD80+ effector Tregs (32.26% ± 2.03% vs 28.68% ± 1.93%; *P* < .05), CD80+ naïve Tregs (1.78% ± 0.33% vs 0.45% ± 0.06%; *P* < .01), CD25 + helper T cells (31.04% ± 1.91% vs 28.70% ± 1.85%; *P* < .05), CD80+ helper T cells (1.53% ± 0.27% vs 0.60% ± 0.08%; *P* < .01), and CD80+ CD8+ (1.11% ± 0.21% vs 0.65% ± 0.12%; *P* < .01).

### 3.5 Differences in the immune cell profile between patients with PD and non‐PD

**Figure 3:**
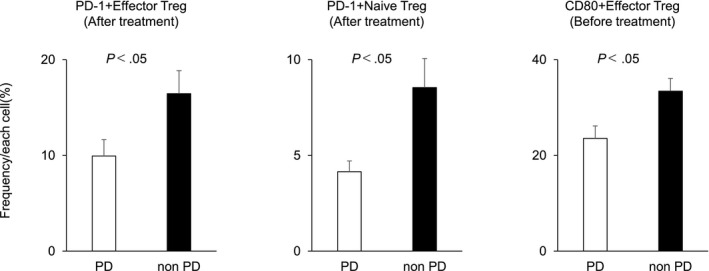
Frequencies of peripheral blood immune cells with each profile in patients in the PD group and non‐PD group. Only significant results are shown. The data are the mean ± SD

**Figure 4:**
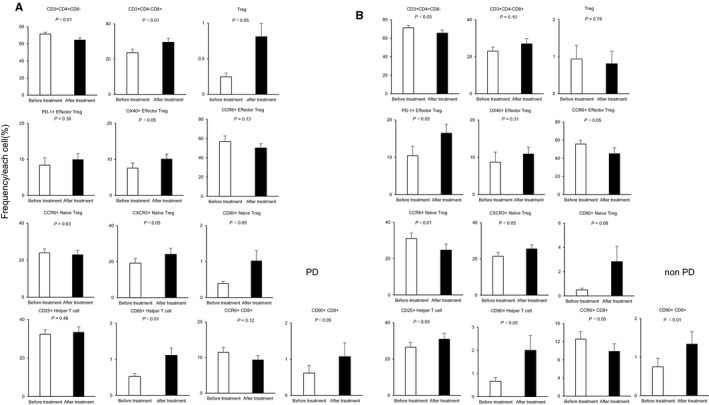
Change of peripheral blood immune cells before and after αβT‐cell therapy. A, The frequencies of peripheral blood immune cells with each profile in patients before αβT‐cell therapy and after αβT‐cell therapy in the PD group. B, The frequencies of peripheral blood immune cells with each profile in patients before αβT‐cell therapy and after αβT‐cell therapy in the non‐PD group. Only significant results are shown. The data are the mean ± SD

A comparison between the PD and non‐PD groups showed that in the non‐PD group, the frequencies of PD‐1+ effector Tregs and PD‐1+ naïve Tregs were significantly higher after treatment, and the frequency of CD80+ effector Tregs was significantly higher before treatment compared with the PD group (PD‐1+ effector Tregs: PD group vs non‐PD group, 9.94% vs 16.45%, *P* < .05; PD‐1+ naïve Tregs: PD group vs non‐PD group, 4.15% vs 8.55%, *P* < .05; CD80+ effector Tregs: PD group vs non‐PD group, 23.55% vs 33.43%, *P* < .05; Figure 3). Figure 4 shows the immune cells for which significantly different frequencies were observed before and after treatment in the PD (Figure 4A) and non‐PD groups (Figure 4B). Those immune cells that exhibited significant changes in frequency in both the PD and non‐PD groups were as follows: CD3+ CD4+CD8– (PD group: 71.38% ± 2.23% [before] vs 64.38% ± 2.56% [after], *P* < .01; non‐PD group: 71.18% ± 2.56% [before] vs 65.46% ± 3.38% [after], *P* < .05); CXCR3+ naïve Tregs (PD group: 19.17% ± 2.65% [before] vs 24.09% ± 3.37% [after], *P* < .05; non‐PD group: 21.50% ± 2.10% [before] vs 25.51% ± 2.28% [after], *P* < .05); CD80+ helper T cells (PD group: 0.52% ± 0.08% [before] vs 1.11% ± 0.21% [after], *P* < .01; non‐PD group: 0.67% ± 0.16% [before] vs 2.00% ± 0.65% [after], *P* < .05); and CD80+ CD8+ (PD group: 0.62% ± 0.21% [before] vs 1.07% ± 0.38% [after], *P* < .05; non‐PD group: 0.75% ± 0.22% [before] vs 1.33% ± 0.32% [after], *P* < .01). Those immune cells for which significant changes were only evident in the PD group were as follows: CD3+CD4‐CD8+ (23.65% ± 1.97% [before] vs 29.58% ± 2.28% [after], *P* < .01); Tregs (0.25% ± 0.05% [before] vs 0.81% ± 0.18% [after], *P* < .05); OX40 + effector Tregs (7.59% ± 1.40% [before] vs 10.06% ± 1.40% [after], *P* < .05); and CD80+ naïve Tregs (0.39% ± 0.06% [before] vs 1.02% ± 0.29% [after], *P* < .05). Conversely, those immune cells for which significant changes were only evident in the non‐PD group were as follows: PD‐1+ effector Tregs (10.42% ± 2.51% [before] vs 16.45% ± 2.40% [after], *P* < .05); CCR6+ effector Tregs (55.75% ± 4.15% [before] vs 45.05% ± 6.60% [after], *P* < .05); CCR6+ naïve Tregs (31.02% ± 3.03% [before] vs 24.68% ± 3.39% [after], *P* < .01); CD25+ helper T cells (26.47% ± 2.63% [before] vs 30.89% ± 3.27% [after], *P* < .05); and CCR6+ CD8+ (12.55% ± 1.69% [before] vs 9.84% ± 1.69% [after], *P* < .05).

### 3.6 Relationship between immune cell profile and prognosis

**Figure 5:**
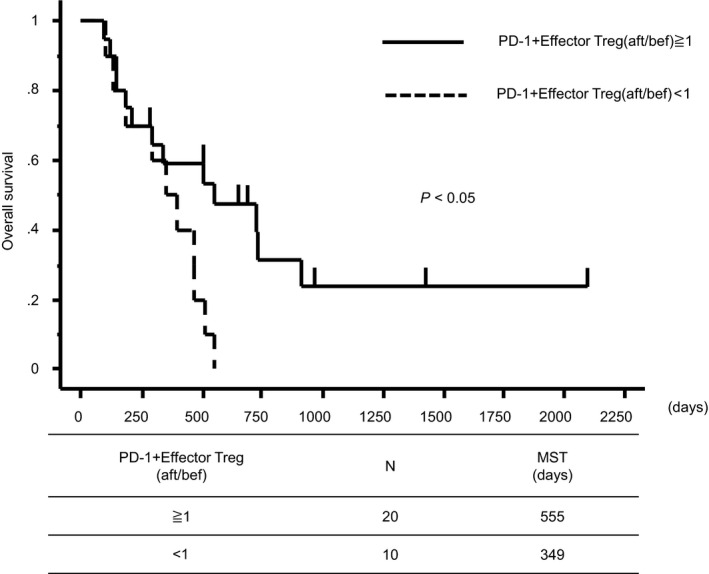
Kaplan‐Meier survival curves for patients in whom the frequency of PD‐1+ effector Tregs increased and decreased after treatment

The 14 immune cell profiles that exhibited significant changes after treatment (Figure 2) were divided into those for which the after/before ratio increased and those for which it decreased, and Kaplan‐Meier survival curves were drawn. Patients in whom the frequencies of PD‐1+ effector Tregs increased had significantly better prognosis than those in whom these immune cells decreased (mean survival time: 555 days [increased group] vs 349 days [decreased group], *P* < .05; Figure 5). No other immune cell profile was associated with a difference in prognosis (Figure S4).

**Figure 6:**
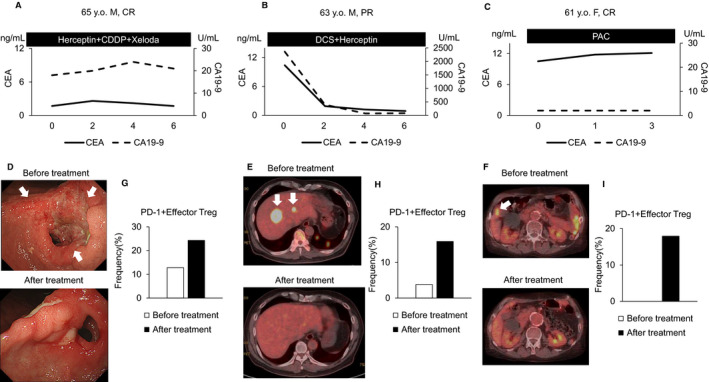
Clinical courses of representable cases of CR and PR. Changes in CEA and CA19‐9 after the start of αβT‐cell therapy (A, B, C). The primary lesion or metastases contracted or disappeared after one course of αβT‐cell therapy (D, E, F). The frequency of PD‐1+ effector Tregs increased in all these patients after treatment (G, H, I)

Figure 6 shows the clinical courses of representative cases of CR and PR in the non‐PD group. In all these patients, the frequency of PD‐1+ effector Tregs increased, and the primary lesion and metastases shrank after treatment (Figure 6A‐I).

### 3.7 Effect of combination chemotherapy on immune cell profiles

Combination chemotherapy was administered to 29 patients. To investigate the effect of combination chemotherapy on their immune cell profiles, we compared those patients who underwent treatment with a platinum‐based regimen (the Platinum group) with those who did not (the other group). A comparison between the Platinum group and the other group showed that in the Platinum group, the frequencies of CCR4+ helper T cells were significantly higher after treatment, and the frequencies of CD14+ MDSCs, CXCR3+ naïve Tregs, and CXCR3+ CD8+ cells were significantly higher before treatment compared with the other group (CCR4+ helper T cells: Platinum group vs other group, 27.51% vs 14.92%, *P* < .05; CD14+ MDSCs: Platinum group vs other group, 21.39% vs 12.76%, *P* < .05; CXCR3+ naïve Tregs: Platinum group vs other group, 25.82% vs 16.33%, *P* < .05; CXCR3+ CD8+: Platinum group vs other group, 83.44% vs 73.11%, *P* < .05). Conversely, in the other group, the frequency of neutrophils was significantly higher after treatment, and the frequency of CD25+ effector Tregs was significantly higher before and after treatment compared with the Platinum group (Neutrophils: Platinum group vs other group, 52.56% vs 67.94%, *P* < .05; CD25+ effector Tregs (before): Platinum group vs other group, 97.54% vs 98.77%, *P* < .05; CD25+ effector Tregs (after): Platinum group vs other group, 96.91% vs 98.26%, *P* < .05; Figure S5).

We compared the immune cell profiles of patients before and after treatment in the Platinum group and the other group (Figure S6). Those immune cells that exhibited significant changes in frequency in both the Platinum and other group were as follows: CD3+ CD4+CD8− (Platinum group: 67.38% ± 2.66% [before] vs 61.94% ± 2.55% [after], *P* < .01; other group: 69.40% ± 2.70% (before) vs 61.70% ± 3.58% [after], *P* < .01); CD3+ CD4‐CD8+ (Platinum group: 26.87% ± 2.41% [before] vs 30.69% ± 2.09% [after], *P* < .05; other group: 25.49% ± 2.54% [before] vs 31.30% ± 3.42% [after], *P* < .01); CD80+ helper T cells (Platinum group: 0.57% ± 0.10% [before] vs 1.59% ± 0.40% [after], *P* < .01; other group: 0.53% ± 0.11% (before) vs 1.19% ± 0.26% [after], *P* < .01); CD80+ CD8+ (Platinum group: 0.61% ± 0.18% [before] vs 0.90% ± 0.27% [after], *P* < .05; other group: 0.67%± 0.18% [before] vs 1.33% ± 0.37% [after], *P* < .05).

Those immune cells for which significant changes were only evident in the Platinum group were as follows: OX40+ helper T cells (1.61% ± 0.45% [before] vs 0.59% ± 0.14% [after], *P* < .05).

Conversely, those immune cells for which significant changes were only evident in the other group were as follows: Tregs (0.44% ± 0.14% [before] vs 1.08% ± 0.29% [after], *P* < .05); PD‐1+ effector Tregs (6.99% ± 1.06% [before] vs 11.68% ± 1.79% [after], *P* < .05); CCR6+ naïve Tregs (27.28% ± 1.47% [before] vs 21.80% ± 2.65% [after], *P* < .05); CXCR3+ naïve Tregs (16.33% ± 2.08% [before] vs 21.40% ± 2.54% [after], *P* < .05); CD80+ naïve Tregs (0.40% ± 0.10% [before] vs 1.13% ± 0.27% [after], *P* < .01); CTLA‐4+ CD8+ (17.79% ± 4.85% [before] vs 16.01% ± 4.53% [after], *P* < .05); CCR6+ CD8+ (11.92% ± 1.47% [before] vs 7.86% ± 1.24% [after], *P* < .01); OX40+ CD8+ (46.30% ± 4.22% [before] vs 43.85% ± 3.64% [after], *P* < .05).

There was no significant difference in survival time between the Platinum group and the other group (mean survival time: 504 days [Platinum group] vs 335 days [other group], *P* = .89; Figure S7).

## 4 DISCUSSION

Our results showed that immune cell profiles changed greatly after αβT‐cell therapy. Although αβT‐cell therapy did not lead to significant changes in the frequency of αβT cells or Tregs, the frequency of CD8+ T cells significantly increased (Figure 2). When we divided patients into the PD group and non‐PD group and investigated the changes after treatment in these groups, we found that the frequency of CD8+ T cells tended to increase in both groups, although this was not significant in the non‐PD group. Tregs increased significantly in the PD group but tended to decrease in the non‐PD group, although the latter difference was not significant (Figure 4). Our results are consistent with that of a previous study that also showed that CD8+ T cells increased and Tregs decreased after αβT‐cell therapy for a range of different advanced cancers, including gastric cancer.^10^ Our results were not inconsistent with previous reports that αβT‐cell therapy improves OS and progression‐free survival by promoting tumor‐directed cytotoxic activity due to increasing the number of CD8+ T cells in peripheral blood, and releasing the immunosuppressive environment by decreasing the frequency of Tregs.^11^, ^12^ The frequency of PD‐1+ effector Tregs also increased after treatment, and this increase was significantly higher in the non‐PD group than in the PD group. The frequencies of PD‐1+ CD4+ T cells and PD‐1+ CD8+ T cells are reportedly significantly higher in gastric cancer patients than in healthy individuals,^13^ and the frequency of PD‐1+ effector Tregs is also known to increase.^14^ Our results suggested that αβT‐cell therapy may encourage PD‐1 expression in effector Tregs.

The post‐treatment increase in PD‐1+ effector Tregs was also associated with extended OS (Figure 5). PD‐1 is expressed by activated immune cells, and is an immune co‐receptor that inhibits T cells that attack autologous tissue and cancer cells.^15^ Similarly, Tregs exhibit high levels of expression of the Forkhead Box P3 (*FoxP3*) gene, regulating self‐reactive immune cells to inhibit autoimmune disease.^16^ Tregs express large amounts of PD‐1, and Tregs isolated from hepatitis C patients and cancer patients exhibit particularly strong PD‐1 expression.^17^, ^18^ Zhang et al investigated the roles of PD‐1 and Tregs in the immune regulation mechanism, and reported that Tregs isolated from PD‐1 knockout mice have a stronger inhibitory effect on autoimmunity than Tregs from wild‐type mice,^19^ indicating that PD‐1 regulates Treg function. Those results suggested that the post‐treatment increase in Tregs with increased PD‐1 expression may work to promote their antitumor effect.

Some advanced gastric cancer patients develop hyperprogressive disease (HPD) after undergoing anti‐PD‐1 monoclonal antibody therapy, with rapid tumor growth occurring.^20^, ^21^, ^22^ Kamada et al investigated changes in the immune profiles of peripheral blood and tumor tissue from advanced gastric cancer patients treated with anti‐PD‐1 monoclonal antibody.^14^ They reported that both the tumors of HPD and non‐HPD patients contain large numbers of effector Tregs, and that among these effector Tregs, CD4+ and CD8+ effector and memory T cells express PD‐1 at around the same level. The use of anti‐PD‐1 antibody and PD‐1 knockout both cause the proliferation of effector Tregs and intensify their antitumor immunosuppressant activity, results not inconsistent with those of Zhang et al Although there was no significant change in the frequency of Tregs after anti‐PD‐1 monoclonal antibody therapy in non‐HPD patients, the ratio of effector Tregs to CD8+ T cells (%) decreased significantly. An association between the ratio of Tregs to CD8+ T cells and prognosis has also been reported for colorectal cancer,^23^ and the predominance of CD8+ T cells over effector Tregs may be associated with improved prognosis.

From our results in this study, we conjectured that αβT‐cell therapy may increase the frequency of PD‐1+ effector Tregs, with PD‐1 weakening the antitumor immunosuppression of effector Tregs, and that αβT‐cell therapy also increases CD8+ T cells, leading to an improved prognosis.

The frequency of CTLA‐4+ effector Tregs also increased significantly after αβT‐cell therapy, although this had no effect on prognosis (Figure 2, Figure S4). This may have been because effector Tregs exhibit higher levels of CTLA‐4 expression than do CD4+ and CD8+ T cells, and that PD‐1+ effector Tregs have higher levels of CTLA‐4 expression than do PD‐1– effector Tregs.^14^

These results suggested that changes in the frequency of PD‐1+ effector Tregs may be a predictive factor for response to αβT‐cell therapy. However, a limitation of this study was that all but one patient underwent αβT‐cell therapy in combination with chemotherapy, and the possibility that this chemotherapy may have affected their immune profiles cannot be excluded. According to Takimoto et al, however, in advanced gastric cancer patients who underwent autolymphocyte therapy or dendritic cell immunotherapy, autolymphocyte therapy improved prognosis irrespective of the use of combination therapy with chemotherapy, surgery, or radiotherapy, and the type of combination chemotherapy also had no effect on their prognosis.^24^ Kamigaki et al also studied the immune profiles of advanced gastric cancer patients after they had undergone immunotherapy, and found that although the majority had previously undergone chemotherapy, their immune profiles changed in different ways depending on the type of immunotherapy.^10^ Our analysis showed that although the effect of combination chemotherapy on the immune cell profile differed depending on the type of combination chemotherapy used, in this study we found no difference in PD‐1+ effector Tregs, which are considered to be associated with improved prognosis (Figure S5), and there was no significant difference in survival time between the two groups (Figure S7). A comparison of the pretreatment and post‐treatment immune cell profiles of patients in the Platinum group and the other group showed that there was no change in PD‐1+ effector Tregs in the Platinum group, but a significant change in PD‐1+ effector Tregs in the other group (Figure S6). These results suggested that the combined use of αβT‐cell therapy may help to improve the prognosis of patients who are not receiving platinum‐based chemotherapy. Accordingly, although it is impossible to say with certainty, we conjectured that it was the changes in immune cell profiles as a result of αβT‐cell therapy that affected the improvement in prognosis seen in this study. However, the effect of combination chemotherapy on immune cell profiles must be borne in mind, and further studies are required.

In this study, we were unable to identify any pretreatment factors predicting response to treatment. However, our comparison of immune cell profiles after the completion of one course with those before the start of treatment revealed whether a significant response to treatment can be achieved, and this may help determine whether or not treatment should be continued.

In conclusion, our results suggested that αβT‐cell therapy changes the host's immune cell profile, and an increase in PD‐1+ effector Tregs may lead to improvement in prognosis.

## CONFLICT OF INTEREST

None.

## AUTHOR CONTRIBUTIONS

Miyabi Miura contributed to study concept, writing of the manuscript, acquisition, analysis and interpretation of data, and final review of the manuscript. Eishiro Mizukoshi contributed to study concept, interpretation of data, critical review, and final review of the manuscript. Tomomi Hashiba and Takafumi Mochizuki contributed to acquisition of data. Masaaki Kitahara, Tomoharu Miyashita, Takafumi Mochizuki, Shigenori Goto, Takashi Kamigaki, Rishu Takimoto, Taro Yamashita, Yoshio Sakai, Tatsuya Yamashita, and Masao Honda contributed to critical review and final review of the manuscript. Shuichi Kaneko contributed to final review of the manuscript.

## Supporting information

Supplementary Material

## Data Availability

All data generated or analyzed during this study are included in this published article and its supplementary information files.
