# Supplementary material for: Effects of adaptive immune cell therapy on the immune cell profile in patients with advanced gastric cancer
Source: Cancer Med. 2020 Jun 11;9(14):4907–17. doi: 10.1002/cam4.3152 (PMC7367616; doi:10.1002/cam4.3152)
Supplement: Supplementary file 1 — Supplementary Material [file CAM4-9-4907-s001.docx]

**Supplementary Figure 1. CONSORT (Consolidated Standards of Reporting Trials) diagram.**

**Supplementary Figure 2. Kaplan-Meier survival curves for the PD and non-PD groups.** Patients in the non-PD group tended to have a better prognosis, although this difference was not significant.

**Supplementary Figure 3. The frequencies of peripheral blood immune cells with each profile in patients before αβT-cell therapy compared to after αβT-cell therapy.** None of these differences were significant. The data are the mean ± standard deviation.

**Supplementary Figure 4. Kaplan-Meier survival curves for immune cell profiles that increased or decreased significantly after treatment.**

**Supplementary Figure 5. The frequencies of peripheral blood immune cells with each profile in patients in the Platinum group and other group.** Only significant results are shown. The data are the mean ± standard deviation.

**Supplementary Figure 6. The change in peripheral blood immune cells before and after αβT-cell therapy.** (a) The frequencies of peripheral blood immune cells with each profile in patients before αβT-cell therapy and after αβT-cell therapy in the Platinum group. (b) The frequencies of peripheral blood immune cells with each profile in patients before αβT-cell therapy and after αβT-cell therapy in the other group. Only significant results are shown. The data are the mean ± standard deviation.

**Supplementary Figure 7. Kaplan-Meier survival curves for the Platinum group and other group.** No significant differences between the Platinum group and other group were found.

Supplementary Table 1. Prior chemotherapy

|  | N |
| --- | --- |
| S-1 | 5 |
| S-1+CDDP | 6 |
| PTX  S-1+DTX | 2  1 |
| S-1+CDDP+LNT | 1 |
| CPT-11  DTX  DTX+5’-DFUR  HER+XP  PTX+RAM  DCS | 1  1  1  1  1  1 |
|  |  |

Abbreviations: S-1, Tegafur/Gimeracil/Oteracil/Potassium; CDDP, cisplatin; DTX, docetaxel; PTX, paclitaxel; CPT-11, irinotecan; LNT, lentinan; 5’-DFUR, doxifluridine; HER, trastuzumab; XP, capecitabine+cisplatin; RAM, ramucirumab; DCS, docetaxel+cisplatin+S-1.

Supplementary Table 2. Combined chemotherapy

|  | N |
| --- | --- |
| S-1+CDDP | 11 |
| PTX | 4 |
| S-1 | 3 |
| CPT-11+CDDP | 2 |
| CPT-11 | 2 |
| SOX | 2 |
| S-1+DTX  HER+Xeloda/PTX  HER+ DCS  HER+XP | 2  1  1  1 |

Abbreviations: S-1, Tegafur/Gimeracil/Oteracil/Potassium; CDDP, cisplatin; PTX, paclitaxel; CPT-11, irinotecan; SOX, S-1+Oxaliplatin; DTX, docetaxel; HER, trastuzumab; Xeloda, capecitabine; DCS, docetaxel+cisplatin+S-1; XP, capecitabine+cisplatin.

Supplementary Table 3. The chemotherapy administered in combination with αβT-cell therapy

|  | N |
| --- | --- |
| First line | 17 |
| Second line | 6 |
| Third line | 2 |
| Fourth line | 1 |
| Fifth line | 3 |

Supplementary Table 4. Adverse events associated with αβT-cell therapy

|  | N |
| --- | --- |
| Fever | 5 |
| Fatigue | 1 |
| Itching | 1 |
| Drowsiness | 1 |

continued
